# Gothenburg Very Early Supported Discharge study (GOTVED): a randomised controlled trial investigating anxiety and overall disability in the first year after stroke

**DOI:** 10.1186/s12883-019-1503-3

**Published:** 2019-11-09

**Authors:** Lena Rafsten, Anna Danielsson, Asa Nordin, Ann Björkdahl, Asa Lundgren-Nilsson, Maria E. H. Larsson, Katharina S. Sunnerhagen

**Affiliations:** 1 Institute of Neuroscience and Physiology, Department of Clinical Neuroscience and Rehabilitation Medicine, Sahlgrenska Academy, University of Gothenburg, Gothenburg, Sweden. Per Dubbsgatan 14, fl. 3, 413 45 Gothenburg, Sweden; 2 Department of Occupational Therapy and Physiotherapy, Sahlgrenska University Hospital, Gothenburg, Sweden; 3 Institute of Neuroscience and Physiology, Department of Health and Rehabilitation, Sahlgrenska Academy, University of Gothenburg, Gothenburg, Sweden; 4 Centre for Person-Centred Care (GPCC), University of Gothenburg, Gothenburg, Sweden; 5 Institute of Social Science, Campus Bräcke, Ersta Sköndal Bräcke University College, Gothenburg, Sweden; 6 Närhälsan, Research and Development Primary Health Care, Gothenburg, Region Västra Götaland Sweden

**Keywords:** Stroke, Very early supported discharge, Anxiety, Rehabilitation

## Abstract

**Background and purpose:**

Early supported discharge (ESD) has been shown to be efficient and safe as part of the stroke care pathway. The best results have been seen with a multidisciplinary team and after mild to moderate stroke. However, how very early supported discharge (VESD) works has not been studied.

The aim of this study was to investigate whether VESD for stroke patients in need of ongoing individualized rehabilitation affects the level of anxiety and overall disability for the patient compared with ordinary discharge routine.

**Methods:**

A randomized controlled trial was performed with intention to treat analyses comparing VESD and ordinary discharge from hospital. All patients admitted at the stroke care unit at Sahlgrenska University Hospital of Gothenburg between August 2011 and April 2016 were screened. Inclusion occurred on day 4 using a block randomization of 20 and with a blinded assessor. Assessments were made 5 days post-stroke and 3 and 12 months post-stroke. Patients in the VESD group underwent continued rehabilitation in their homes with a multidisciplinary team from the stroke care unit for a maximum of 1 month. The patients in the control group had support as usual after discharge when needed such as home care service and outpatient rehabilitation.

The primary outcome was anxiety as assessed by the Hospital Anxiety and Depression Scale-Anxiety subscale (HADS-A). The secondary outcome was the patients’ degree of overall disability, measured by the modified Rankin Scale (mRS).

**Results:**

No significant differences were found between the groups regarding anxiety at three or 12 months post-stroke (*p* = 0.811). The overall disability was significantly lower in the VESD group 3 months post-stroke (*p =* 0.004*)*, compared to the control group. However, there was no significant difference between the groups 1 year post-stroke.

**Conclusions:**

The VESD does not affects the level of anxiety compared to ordinary rehabilitation. The VESD leads to a faster improvement of overall disability compared to ordinary rehabilitation. We suggest considering coordinated VESD for patients with mild to moderate stroke in addition to ordinary rehabilitation as part of the service from a stroke unit.

**Trial registration:**

Clinical Trials.gov: NCT01622205. Registered 19 June 2012 (retrospectively registered).

## Background

Physical impairments are often present after a stroke, as are a variety of psychological consequences, for instance mood disorders, which can compromise the rehabilitation process and influence long-term recovery [1]. Globally the most common mental health problems post-stroke are anxiety disorders [2], generally reported by 3.8–25% of patients post-stroke [3]. Anxiety disorders refer to a group of mental disorders characterized by feelings of anxiety and fear including generalised anxiety disorder, panic disorder, phobias and social anxiety disorder [4]. As with depression, symptoms can range from mild to severe. Anxiety is a common symptom after stroke onset, both in the acute phase and in the chronic phase [5–7]. Approximately 29% suffer from anxiety during the first year after stroke [8]. A review from 2012 concluded that anxiety after stroke receives significantly less attention compared to other psychological problems after stroke [9].

Early supported discharge (ESD) with continued rehabilitation in the home from a multidisciplinary stroke team has been shown to be beneficial [10]. This form of rehabilitation can accelerate the discharge from the hospital [11, 12]. The activity in daily life (ADL) ability was found to be the same after this form of rehabilitation as after inpatient rehabilitation [13, 14]. This type of care can reduce activity impairment and increase independent living and patient satisfaction compared to conventional care [15]. A permanent team including a nurse, a physiotherapist and an occupational therapist is necessary for efficacy [16]. At the time of this study ESD was not implemented in Sweden as a standard rehabilitation option. Despite the fact that today ESD is recommended in national stroke guidelines, it is still not fully implemented and it is unclear how many hospitals in Sweden today offer this form of rehabilitation.

Today, many patients in Sweden are being discharged home very early after stroke. According to the Swedish stroke quality register, the average hospital stay in Sweden after acute stroke is 13 days (median 7 days) [17]. This is much shorter than in the referred studies investigating ESD [10, 18]. We are using the term very early supported discharge (VESD) in the current study due to the shortened hospital stay in Sweden the last years.

There are no apparent differences seen in mood scores such as anxiety between ESD groups and groups receiving ordinary rehabilitation [10], but it is unknown whether VESD influences the level of anxiety during the first year after stroke. The assumption is that some anxiety is normal when being discharged very early after stroke [5–7], but with the supported discharge intervention, one can perhaps reduce the risk of anxiety during the first year after stroke.

The primary aim of the present study was to investigate whether VESD with continued rehabilitation from a multi-professional stroke team from the stroke unit affects the level of anxiety compared to ordinary discharge routine. A secondary aim was to evaluate whether VESD is useful regarding overall disability for stroke patients in need of ongoing individualised rehabilitation at home due to motor and/or cognitive impairment.

## Methods

### Study design

The Gothenburg Very Early Supported Discharge study (GOTVED) is a randomised controlled trial with blinded assessors. This trial is registered on clinicaltrials.gov (identifier: NCT01622205). Participants were enrolled in the trial from September 2011 to April 2016.

### Study population

**Fig. 1:**
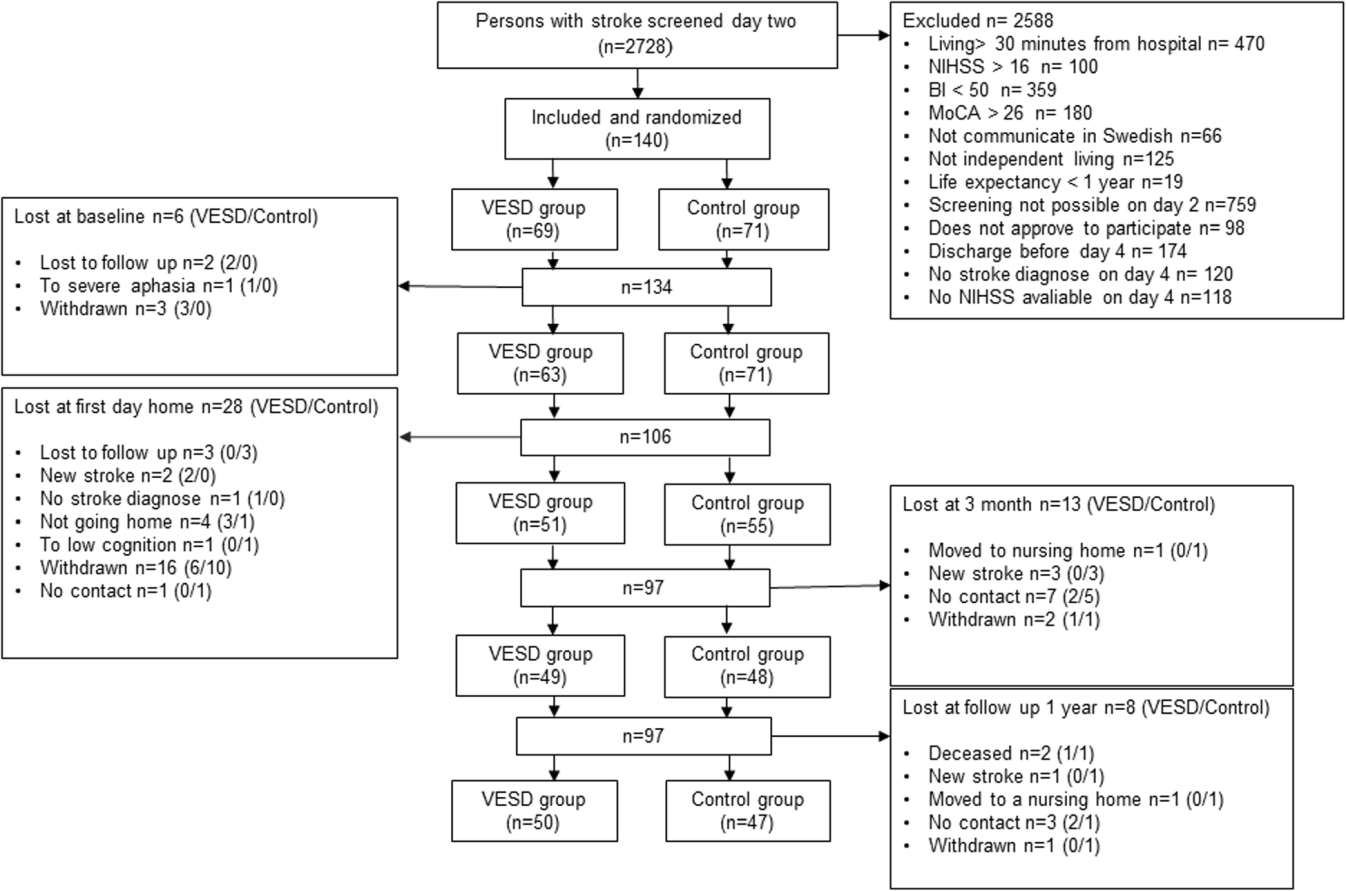
Flow chart of patients allocated to the GOTVED study VESD: very early supported discharge, NIHSS: National Institute of Health Stroke Scale, BI: Barthel Index, MoCA: Montreal Cognitive Assessment, HADS-A: Hospital Anxiety and Depression Scale-Anxiety subscale, mRS: modified Rankin Scale

All patients admitted to one of the stroke care units at Sahlgrenska University Hospital of Gothenburg were consecutively screened (Fig. 1). Patients fulfilling the inclusion criteria were informed by a research coordinator about the study and asked if they wanted to participate. Written informed consent was obtained from the participants or from their closest relative. A block randomization model was applied (20 patients in each block; 10 in the VESD group and 10 in the control group). A person not otherwise involved in the study performed the allocation. This was done by putting a paper slip with group information in envelopes, then sealing, mixing and numbering them. The research coordinator opened an envelope after inclusion, and then the blinded assessor and the nurse in the stroke team were informed of the inclusion. The blinded assessor worked at a different ward at the hospital to minimise learning of the allocation by chance. The CONSORT checklist was followed. The stroke subtypes were confirmed by imaging, and treatment of thrombolysis or thrombectomy was recorded.

Inclusion criteria were confirmed stroke according to World Health Organization criteria [19, 20], age > 18 years, residence within 30 min by car of the stroke unit, a National Institute of Health Stroke Scale (NIHSS) [21] score of 0–16 points, which corresponds to mild to moderate stroke [20], a Barthel Index (BI) [22] score of > 50 points on day two [23], and a Montreal Cognitive Assessment (MoCA) [24] index of < 26 if BI = 100. Thus, exclusion criteria were a NIHSS score of > 16 and a BI score < 50. Patients with a life expectancy of < 1 year (e.g. with severe malignancy) or who were unable to speak or communicate in Swedish prior to stroke were also excluded.

### Outcome measures

The primary outcome as stated in the protocol was anxiety [25]. The anxiety was assessed with the Hospital Anxiety and Depression Scale-Anxiety subscale (HADS-A) [26], which was administered 5 days (± 1 day), 3 months (± 3 days), and 12 months (± 1 week) post-stroke. HADS is a 14-item self-assessment scale that can be divided into two equal parts, HADS-A for anxiety (score 0–21) and HADS-D for depression (score 0–21). A higher score indicates more symptoms of anxiety or depression [27]. The secondary outcome was the patients’ degree of overall disability, measured by the modified Rankin Scale (mRS) [28]. The scale runs from 0 to 6, with zero indicating perfect health without symptoms and six indicating death. The scores can be dichotomised, and a score of ≤2 indicates functional independence [29]. ADL function was assessed by the BI, which ranges from 0 to 100, with higher scores indicating a higher level of ADL independence [30]. Cognitive functions were assessed with the Montreal Cognitive Assessment (MoCA) [24] with a score of ≥26 indicating normal cognitive functioning [31]. Stroke-related neurological deficits were assessed with the National Institute of Stroke Scale (NIHSS) [21] by the nurse at the stroke unit during the first 2 days; the value at day two was used for inclusion. BI and MoCA were administered by occupational therapists 36–48 h after arrival at the stroke unit. A trained, blinded researcher not working at the stroke unit performed the HADS-A and mRS assessments at baseline and all assessments at three and 12 months post-stroke. Exact time points for all assessments are listed in Table 1.

**Table 1:** Assessments

Assessment				
	Day 2	Day 5	Month 3	Month 12
NIHSS	x			
BI	x		x	x
MoCA	x		x	x
HADS		x	x	x
mRS		x	x	x

*NIHSS* National Institute of Health Stroke Scale, *BI* Barthel Index, *MoCA* Montreal Cognitive Assessment, *HADS* Hospital Anxiety and Depression Scale, *mRS* modified Rankin Scale

### Intervention in the very early supported discharge group

Prior to discharge of the patients in the VESD group, as a part of the person-centred intervention, a goal-setting meeting was held at which the patient was asked to formulate his or her goals based on the Canadian Occupational Performance Measure [32]. These goals guided the focus of the rehabilitation. Examples of goals were to be able to go to the local store to buy milk, to be able to hang the laundry, or to be able to travel on the tram to a daughter, or to manage the bills. The intervention had a person-centred approach based on the person, her or his context, history, next of kin, individual strengths and weaknesses and expressed personal goals [33].

A rehabilitation team made up of physiotherapists, occupational therapists, and a stroke nurse from the stroke care unit continued the rehabilitation in the patient’s home. At discharge, the intervention group received an individual schedule for the first week of home rehabilitation. The intervention comprised 2–4 visits per week by the physiotherapist and/or occupational therapist and 1–2 visits by the stroke nurse. The intervention could include varied activities or methods to think about different ways of adapting in difficult situations. For some patients, the intervention was to try the intended activities with safe support so that they could feel secure in their performance. The patient and the VESD team together decided when the support from the team should end; the maximum length was 4 weeks after discharge. In connection with the discharge from the VESD, if necessary, the patients were referred to outpatient rehabilitation that would carry on the rehabilitation afterwards. Information and support to next of kin and the home care service on how to best support the patient to reach the decided goals was also of importance [25].

### Control group

Patients in the control group were discharged according to the department’s usual routine. They had no goal-setting meeting and were not followed up by a multidisciplinary team from the stroke unit. However when needed, they were referred to continued rehabilitation or/and support (e.g. outpatient rehabilitation with a physiotherapist and/or an occupational therapist, home care service).

### Statistical analyses

A power calculation was performed based on the level of anxiety (assessed with the HADS) [25]. With a power of 80% and a *p*-value of 0.05 (2-sided test), a sample size of 44 patients per group were needed to detect a 4 point difference [34, 35]. As deaths may occur or consent be withdrawn, we aimed at 55 patients per group. During the inclusion process, more participants than expected dropped out. Therefore, after 2 years of screening, we decided to include a total of 140 patients. Intention to treat analyses were performed, and for missing observations, the “last value carried forward” was used meaning that everyone who started the study was included in the analysis, even though they did not complete the treatment. This meant that dropouts were also included in the analysis. The HADS outcome is presented both as a continuous variable and as a trichotomized. Shifts in the proportions of anxiety disorders were analysed for the intervention group and control group at admittance and after three and 12 months and reported using bar graphs. For this analysis the HADS-A scores at baseline and after three and 12 months were trichotomized according to the subscale scores proposed in the assessment tool: “no annoying anxiety” (0–7) = 1, “mild to moderate anxiety” [8–10]=2, and “occurrence of anxiety disorders” (> 10) = 3 [26]. This was also performed with the mRS: 0 = no symptom at all, 1 = no significant disability despite symptoms, 2 = slight disability, 3 = moderate disability, 4 = moderately severe disability. For each outcome, the common odds ratio and 95% confidence intervals were reported for the shift in the direction of a better outcome in both groups. The chi-square test and Mann-Whitney U-test were used to test for group differences in descriptive data, and to evaluate whether there was any statistically significant difference in outcome between those who were included in the study and those who were not. Descriptive statistics are expressed in percentages, mean ± SD or median and interquartile range (IQR), as appropriate. The Wilcoxon signed rank test was used to assess the change in proportions between admittance, 3 months and 12 months after onset with the HADS-A and the mRS. The effect size was reported according to Cohen [36]. A two-sided value of *p* ≤ 0.05 was considered statistically significant. Statistical analyses were performed using IBM SPSS statistics for Windows, version 24.0 (IBM Corp., Armonk, NY, USA).

## Results

Overall, a total of 2728 patients admitted to the stroke unit at Sahlgrenska University Hospital in Gothenburg were screened. Of these, 140 patients with haemorrhagic or ischaemic stroke were included in the study. Unfortunately, one patient was randomly allocated in error; therefore, 69 patients were randomized to the VESD group and 71 to the control group. The most common cause of exclusion was that the patients could not be assessed with the BI and the MoCA on day 2 (36–48 h after admittance) (Fig. 1). Reasons for this included that the patient was unavailable due to many daytime clinical examinations, or the 36–48 h interval occurred on a Sunday when the assessors did not work. Descriptive statistics and a comparison of the characteristics of the population are given in Table 2. Stroke severity at admittance was mild, with a median NIHSS score of 2. The intention to treat analysis was performed on 140 patients, of which 50 (72%) in the intervention group and 47 (66%) in the control group remained throughout the whole study period. All patients who were lost to follow up because of not being able to get in contact were invited to the next follow-up. There was no statistically significant difference in age, sex or NIHSS at baseline between the intervention and control groups or between those who were included in the study and those who were not. Motor function was assessed with Fugl Meyer Assessment Scale Upper Extremity (UE) (0–66) and Lower Extremity (LE) (0–34) for a descriptive purpose and there was no significant difference between the groups, nor at baseline, or 1 year post-stroke.

**Table 2:** Baseline demographics and clinical characteristics

	All (*n* = 140)	VESD group (*n* = 69)	Control group (*n* = 71)	*p*
Age (years)				
Mean (SD)	74.1 (11.8)	75.5 (11.1)	72.7 (12.4)	*0.17*
Median (IQR)	76 (68–82.75)	78 (69.5–84)	75 (65–82)	
Male (%)	86 (61.4)	42 (60.9)	44 (62)	*0.01*
Length of hospital stay (days)				
Mean (SD)	12.8 (7.7)	11.8 (6.7)	13.8 (8.4)	*0.17*
Median (IQR)	11.0 (8–16)	10.0 (8–15.5)	12.0 (8–16)	
Stroke subtype, *n (%)*				
Ischemic infarct/ Intracerebral haemorrhage	130 (92.8)/ 10 (7.1)	66 (95.6)/ 3 (4.3)	64 (90.1)/ 7 (9.9)	*0.22*
Treatment, *n (%)*				
Thrombolysis/Thrombectomy	15 (10.7)/6 (4.3)	6 (8.7/4 (4.3))	9 (12.7)/3 (4.2)	
NIHSS^a^, Median (IQR)	2 (0–4), *n = 139*	2 (1–4.5)	3 (1–5.25), *n = 70*	*0.02*
BI^a^, Median (IQR)	80 (65–90), *n = 139*	82.5 (65–90), *n = 68*	80 (65–90)	
MoCA^a^, Median (IQR)	22 (19–26), *n = 105*	23 (20–26), *n = 57*	22 (18–25.7), *n = 48*	*0.05*
HADS-A Median (IQR)	4 (1–8), *n = 134*	4 (1–8), *n = 63*	4 (1–8)	*0.22*
HADS-D Median (IQR)	3 (1–7), *n = 134*	3 (1–6), *n = 63*	3 (1–7)	*0.53*
mRS Median (IQR)	2 (2–3), *n = 134*	2 (2–3), *n = 63*	2 (2–3)	*0.14*

*VESD* very early supported discharge, *SD* standard deviation, *IQR* inter quartil range, *NIHSS* National Institute of Health Stroke Scale, *BI* Barthel Index, *MoCA* Montreal Cognitive Assessment, *HADS-A* Hospital Anxiety and Depression Scale-Anxiety subscale, *HADS-D* Hospital Anxiety and Depression Scale-Depression subscale, *mRS* modified Rankin Scale

^a^Second day (36–48 h) after arrival to stroke unit

The patients in the VESD group received a median of 11 (IQR 7–14) visits from the team over 4 weeks, and each visit lasted an average of about 1 h. The most common rehabilitation treatment in the VESD group was to improve ADL, in order to be able to manage daily activities in one’s home, to be able to walk in a more secure way indoors and outside and to be able to travel by public transportation. After discharge from the VESD team 58 % received continued rehabilitation during the first year after stroke onset. In average the intervention group received nine visits during the first 3 month after onset and six visits during the first year.

Seventy-six percent (54) of the patients in the control group were referred to some sort of continued rehabilitation after discharge from the stroke unit, such as a rehabilitation unit, primary care, or community care. Of those, six continued inpatient rehabilitation and stayed there for an average of 31 days (IQR 10–56). After discharge 50 patients in the control group received continued outpatient rehabilitation during the first year after stroke onset such as physiotherapy, occupational therapy and/or speech and language therapy. In average, they received seven visits per patient during the first 3 months after onset, and 13 visits during the first year post stroke.

**Fig. 2:**
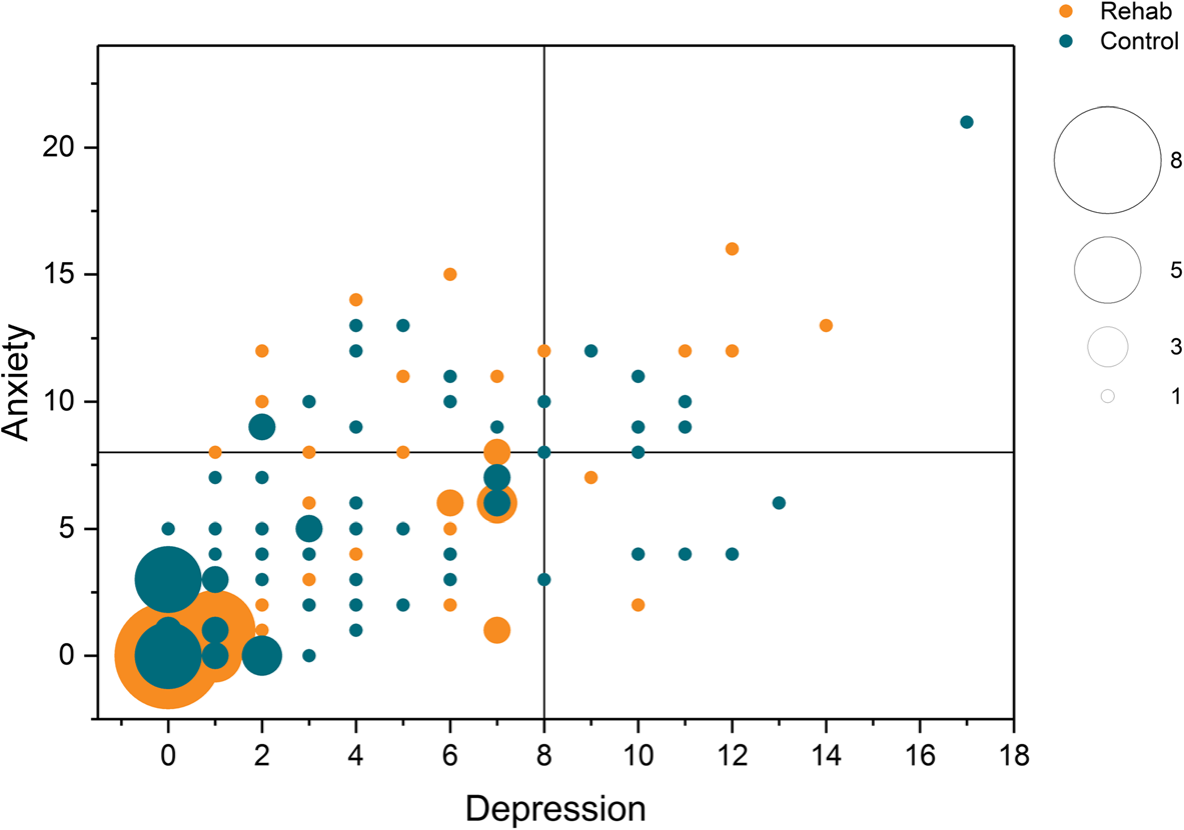
Scatterplot anxiety and depression at baseline

There was no significant difference at baseline between the groups regarding HADS-A. Three months after onset, there was a significant difference between the groups regarding HADS-A (Table 3) were the VESD group had a decreased level of anxiety compared to the control group (*p* = 0.05). Of those having some form of self-assessed anxiety at baseline, 10% also had self-assessed depression (Fig. 2). We could not show any significant difference at baseline regarding mRS, but 3 months post-stroke the mRS was significantly lower in the VESD group compared to the control group (*p =* 0.004*)*. One year post-stroke there was no significant difference between the groups (Table 3). There were no unintended effects or harmful incidents reported regarding anxiety or degree of overall disability in the groups.

**Table 3:** Comparison of outcome variables across groups

Assessment	All (*n* = 140)		VESD group (*n* = 69)		Control group (*n* = 71)		*p* m3	*p* m 12
	m3	m12	m3	m12	m3	m12		
BI, Median (IQR)	100 (90–100)	100 (86–100)	S	100 (90–100)	100 (90–100)	100 (85–100)	*0.22*	*0.76*
HADS-A								
Median (IQR)	3 (0–7), *n = 134*	3 (0–7), *n = 134*	2 (0–6), *n = 63*	3 (0–7), *n = 63*	4 (1–7)	4 (1–7)	0.05*	*0.48*
% ≥8**	21	21	21	22	21	20		
HADS-D:								
Median (IQR)	1 (1–1) *n = 134*	1 (1–1) *n = 134*	1 (1–1) *n = 63*	1 (1–1) *n = 63*	1 (1–1)	1 (1–1)	*0.17*	*0.58*
% ≥ 8	14	14	10	10	18	17		
mRS, Median (IQR)	2 (1–3)	2 (1–3)	2 (1–2), *n = 63*	2 (1–3), *n = 63*	2 (2–3)	2 (1–3)	≤0.01*	*0.08*
MoCA, Median (IQR)	24 (20–26)	24 (21–26)	24 (20–26), *n = 66*	24 (20–26), *n = 66*	24 (19–26), *n = 63*	24 (21–26), *n = 64*	*0.43*	*0.79*

*VESD* very early supported discharge, *m* months, *IQR* inter quartile range, *NIHSS* National Institute of Health Stroke Scale, *BI* Barthel Index, *MoCA* Montreal Cognitive Assessment, *HADS-A* Hospital Anxiety and Depression Scale-Anxiety subscale, *mRS* modified Rankin Scale

*Statistically significant (*p* ≤ 0.05), **Percent with anxiety or depression, HADS-A ≥ 8, HADS-D ≥ 8

**Fig. 3:**
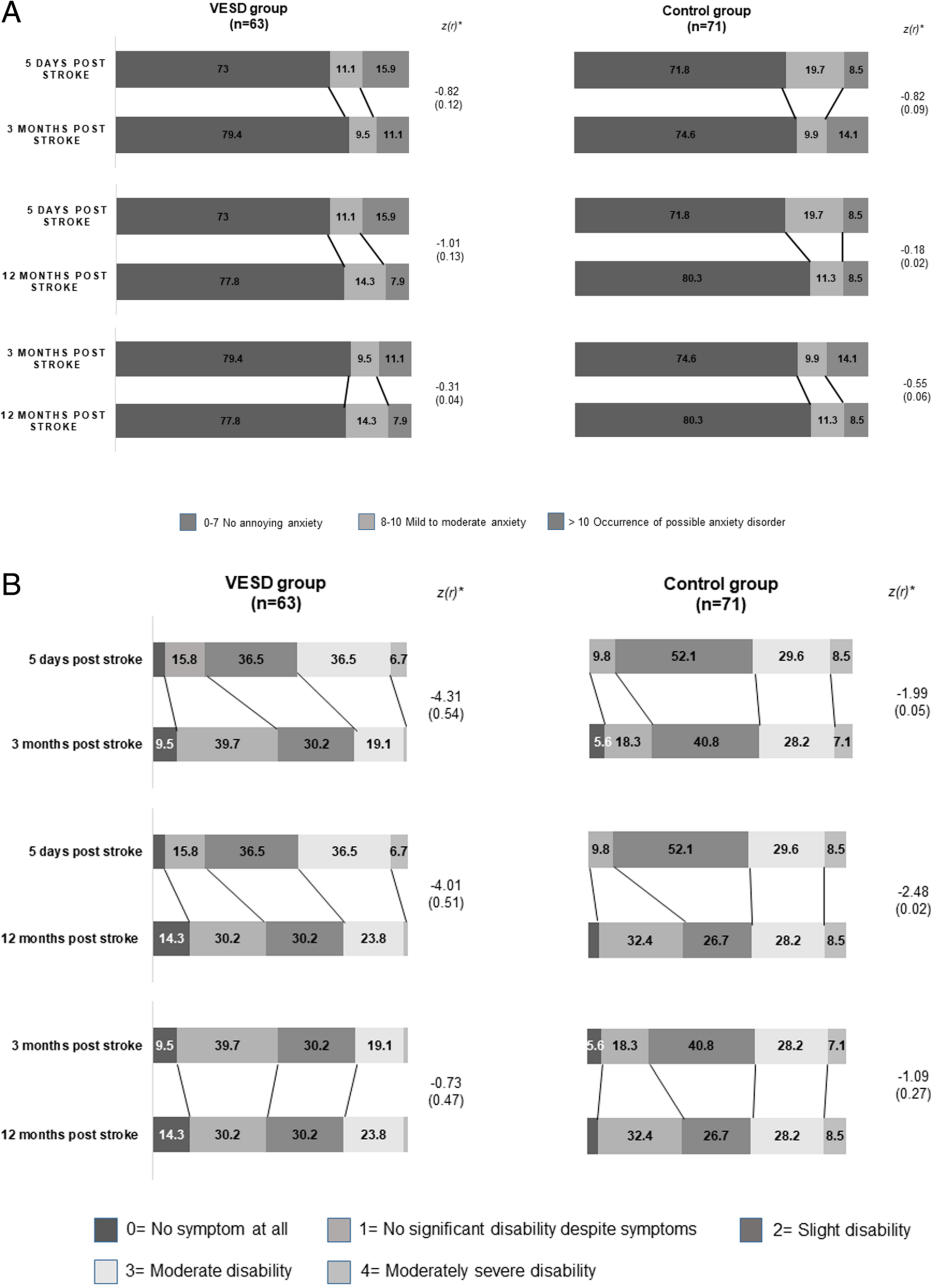
**a** Shift in the proportions (%) of levels of anxiety assessed with HADS-A between admittance and after three and 12 months post stroke. Values ≤5% are not specified in the figure. *Results of the Wilcoxon signed rank test between admittance, 3 months and 12 months post stroke VESD: very early supported discharge, HADS-A: Hospital Anxiety and Depression Scale-Anxiety subscale. **b** Shift in proportions (%) of levels of degree of overall disability assessed with mRS between admittance and after three and 12 months post stroke. Values ≤5% are not specified in the figure. *Results of the Wilcoxon signed rank test between admittance, 3 months and 12 months post stroke VESD: very early supported discharge, mRS: modified Rankin Scale

The shift in the proportions of the trichotomized HADS-A and mRS scores, divided into seven domains, is illustrated in Fig. 3. The shift in the proportion of HADS only showed a small effect size, where the shift went against reduced anxiety, in both the VESD group and the control group in all analyses (Fig. 3a). In the VESD group, there was a clearer change in proportions for mRS between admittance and 3 months post-stroke, with a large effect size (*r* = 0.54, *p* < 0.001), and between admittance and 12 months post-stroke (*r* = 0.51, *p* < 0.001). Between 3 and 12 months, there was a medium effect (*r* = 0.47, *p* = 0.47). In the control group, there were only small effect sizes in the shift of proportions in mRS (Fig. 3b).

## Discussion

Our aim was to assess the presence and severity of any anxiety in patients after stroke, depending on what sort of rehabilitation they received. We did not have the intention to diagnose anxiety. We could not show any difference in anxiety related to group allocation. The presence of anxiety in our study agreed well with earlier studies, in which approximately 24% of stroke patients were reported to experience anxiety of varying degrees between 3 and 12 months post-stroke [8, 37].

Another finding was that VESD can accelerate the recovery of mild to moderate stroke survivors, as measured by the degree of overall disability using the mRS. It may be possible to reduce disability, at least for a specific group of patients with stroke. These results are in line with previous results for early supported discharge [10]. A possible explanation for this is the dosage. Not all (46%) in the control group received continued outpatient rehabilitation. The control group had an average of three visits compared with five visits in the VESD group during the first 3 months after stroke onset. This can have an impact on the mRS and this may be an explanation for the fact that the VESD group had a lower mRS value during the three-month follow-up.

For this study, we screened > 2700 patients to be able to include 140 patients in the study. This was due to having very low numbers that matched the inclusion criteria (5%) compared to previous reviews [10]. This may be due to some of our inclusion criteria. One of the inclusion criteria was NIHSS ≤16 on day two, which corresponds to mild to moderate stroke [20]. The participants had a median value of NIHSS = 2 on the second day, but at discharge from the hospital, the NIHSS score was often zero, indicating normal neurological function. The median BI value on day two in the VESD group was 82.5. Kay et al. concluded that BI ≤80 is the optimal cut-off for self-reported dependency [23]. A score of 75–90 is reported as mild dependency and 50–74 as moderate dependency [38]. We wanted to include patients with mild to moderate stroke, but we mainly captured those with mild stroke. With a somewhat higher NIHSS score and lower BI values as inclusion criteria, we perhaps would have captured not only patients with mild stroke but also more patients with moderate stroke, and the exclusion rate would have been lower.

Our intention was to give support and make it possible to discharge patients very early compared to early, as in other studies on ESD. Therefore, we made our inclusion decisions based on values from day two, whereas some of the previous studies made them based on values from day 4. Perhaps screening on day four instead of day two would have captured more patients with mild to moderate stroke. The problems with the inclusion criteria for ESD have also been noted in other studies [39]. Further studies are needed to investigate both the optimal inclusion criteria and the best day for screening in order to be able to offer this form of rehabilitation to as many patients as possible who are post-stroke and in need of continued rehabilitation.

Our results show that, although the VESD group was discharged 2 days earlier than the control group, there was no significant difference between the groups regarding length of hospital stay. We had an average length of stay at the stroke unit of 12 days in the VESD group and 14 days in the control group. The finding that there was a difference of only 2 days, compared to earlier studies, could be explained by the overall reduction in the length of hospital stay of all stroke patients in the last few years, as shown in a Cochrane review from 2017 [10]. Therefore, a reduction in the length of hospital stay at very early discharge is probably overly optimistic.

Strengths of the current study are that power was calculated before the start of the study, the randomization was concealed and the assessor was blinded. It is also a strength that the intervention was given by a multidisciplinary team coordinated from the stroke unit, as shown in previous research [16]. Another strength is that the intervention did not aim to influence the procedures at the stroke unit, only to provide the possibility for early supported discharge. That the decision, however, was with physicians not involved in the study. It is also a strength that both groups were similar at baseline.

A limitation with this study is that the set level for NIHSS and BI scores could have an impact on enrolment and that the screening was performed mainly on weekdays. Although the stroke unit was aware of the intervention group receiving very early supported discharge, this did not seem to influence the discharge very much, which resulted in rather long hospital stays in the intervention group, as well. A third limitation was that the choice of inclusion window may have impacted the fact that we mainly captured patients with mild stroke due to our intention to capture patients with mild to moderate stroke. A limitation is that the study cannot distinguish pre-existing psychiatric symptoms from problems caused by the current cerebrovascular accident, as the premorbid emotional status is unknown.

Our study did not find any significant difference in anxiety after stroke depending on when and how the patient was discharged. Therefore, one should not be doubtful from a mood perspective in discharging patients with stroke very early. A previous study found that the change and disruption of life becomes more clear for the patient once at home [40]. This method can make the patient more aware of and more motivated for rehabilitation. This may be one explanation for the significantly lower mRS score in the VESD group compared to the control group 3 months post-stroke. The impact of stroke severity on anxiety is unknown and could have impacted the results in this study.

## Conclusions

- Very early supported discharge after stroke does not affect the level of anxiety at any point compared to ordinary rehabilitation.
- Very early supported discharge leads to a faster recovery of independence after discharge.
- We suggest that coordinated very early supported discharge could be considered for patients with mild to moderate stroke, in addition to ordinary rehabilitation, as a part of the service from a stroke unit.

## Data Availability

The datasets used and analysed during the current study are available from the corresponding author on reasonable request.

## Abbreviations

ADL: Activity of Daily Life
BI: Barthel Index
CI: Confidence interval
ESD: Early Supported Discharge
GOTVED: Gothenburg Very Early Supported Discharge
HADS: Hospital Anxiety and Depression Scale
IQR: Interquartile range
MoCA: Montreal Cognetive Assessment
mRS: modified Ranking Scale
NIHSS: National Institute of Health Stroke Scale
OR: ODDS ratio
VESD: Very Early Supported Discharge
